# Phosphatase of Regenerating Liver-3 Localizes to Cyto-Membrane and Is Required for B16F1 Melanoma Cell Metastasis *In Vitro* and *In Vivo*

**DOI:** 10.1371/journal.pone.0004450

**Published:** 2009-02-13

**Authors:** Ran Song, Feng Qian, Yu-Pei Li, Xia Sheng, Shao-Xian Cao, Qiang Xu

**Affiliations:** State Key Laboratory of Pharmaceutical Biotechnology, School of Life Sciences, Nanjing University, Nanjing, China; Universität Heidelberg, Germany

## Abstract

**Background:**

Phosphatase of regenerating liver-3 (PRL-3) is a member of the novel phosphatases of regenerating liver family, characterized by one protein tyrosine phosphatase active domain and a C-terminal prenylation (CCVM) motif. Though widely proposed to facilitate metastasis in many cancer types, PRL-3's cellular localization and the function of its CCVM motif in metastatic process remain unknown.

**Methodology/Principal Findings:**

In the present study, a series of Myc tagged PRL-3 wild type or mutant plasmids were expressed in B16F1 melanoma cells to investigate the relationship between PRL-3's cellular localization and metastasis. With immuno-fluorescence microcopy and cell adhesion/migration assay *in vitro*, and an experimental passive metastasis model *in vivo*, we found that CCVM motif is critical for the localization of PRL-3 on cell plasma membrane and the lung metastasis of melanoma. In particular, Cystine170 is the key site for prenylation in this process.

**Conclusions/Significance:**

These results suggest that cellular localization of PRL-3 is highly correlated with its function in tumor metastasis, and inhibition of PRL-3 prenylation might be a new approach to cancer therapy.

## Introduction

Phosphatase of regenerating liver (PRL)-3 is a phosphatase with small molecular weight (22 kDa). Recently, this member of the PRL family has been found to play a critical role in the processes of tumor initiation and development, such as cancer cell invasion and migration, tumor angiogenesis and metastasis [1]–[4]. PRL-3 has also been proposed as a biomarker for advanced malignancy in gastric cancer, ovarian cancer, colorectal carcinoma etc. and implicates poor clinical outcome [5], [6]. The potential great importance of PRL-3 in malignant tumors could also be supported from research insights into its structure.

The three members of PRL class share at least 75% amino acid sequence similarity [7]–[9]. With the other two members (PRL-1 and PRL-2), PRL-3 shares the family's common structure of two functional domains. The catalytic domain constitutes one critical motif for PRL-3 and functions as a protein tyrosine phosphatase (PTP) for dephosphorylation similar to most other dual phosphatases [8]. Though its specific substrate *in vivo* has not been clearly identified yet, a number of papers have addressed the importance of this PTP domain of PRL-3 or other proteins in promoting cancer cell growth, invasion and metastasis [10], [11]. Our previous research also indicated that PRL-3 with inactive PTP domain reduced the migratory ability of tumor cells [3]. The other functional domain of PRL-3 is the CCVM motif for prenylation. In contrast to the known effects of the PTP domain as mentioned above, it is unknown if the CCVM motif contributes to the progress of cancer metastasis.

Prenylation is a common post-translational modification for proteins that are targeted to membranes or other interacting factors [12]. The CAAX sequence on the C-terminal of PRL-3 constitutes a conserved feature of this kind of protein family. The recognition of prenylation motifs such as CAAX, XXCC, XCXC and CCXX by farnesyltransferase (FT) or geranylgeranyltransferase (GGT), aids in the correct localization of a variety of proteins to specific sites within the cell and enables participation in their relevant signal transduction pathways [13], [14]. Prominent members of the family such as Ras and Rab, including H-Ras, K-Ras and N-Ras, are prenylated resulting in targeting to plasma membrane or Golgi for their cellular function in tumor proliferation and anti-apoptosis. Likewise, Rho-A, Rho-B and Rho-C localize to plasma membrane after this prenylation resulting in promotion of tumor metastasis and angiogenesis [12], [15], [16].

There is no current consensus on the subcellular localization of PRL-3. Zeng et al. [17] reported that PRL-1, -2, and -3 were targeted to plasma membrane and the early endosomes while a small fraction of unprenylated molecules were localized in the nucleus. In contrast other researchers such as Fiordalisi et al. [18] demonstrated that PRL-3 localized in endomembranes. Since cellular localization could establish a causal link with the molecular intracellular function, the location of PRL-3 needs to be further elucidated. Therefore, here we explored the role of prenylation of the CCVM motif in PRL-3's cellular localization and in the process of tumor cell metastasis. Consistent with our recent work demonstrating the important role of PRL-3 in melanoma metastasis [3], [19], the data presented here offer new insight to the functional role of PRL-3 stressing the importance of subcellular localization in the regulation of metastasis.

## Results

### Construction of Myc-PRL-3 fusion expression vector and expression in B16F1 murine melanoma cells

**Figure 1:**
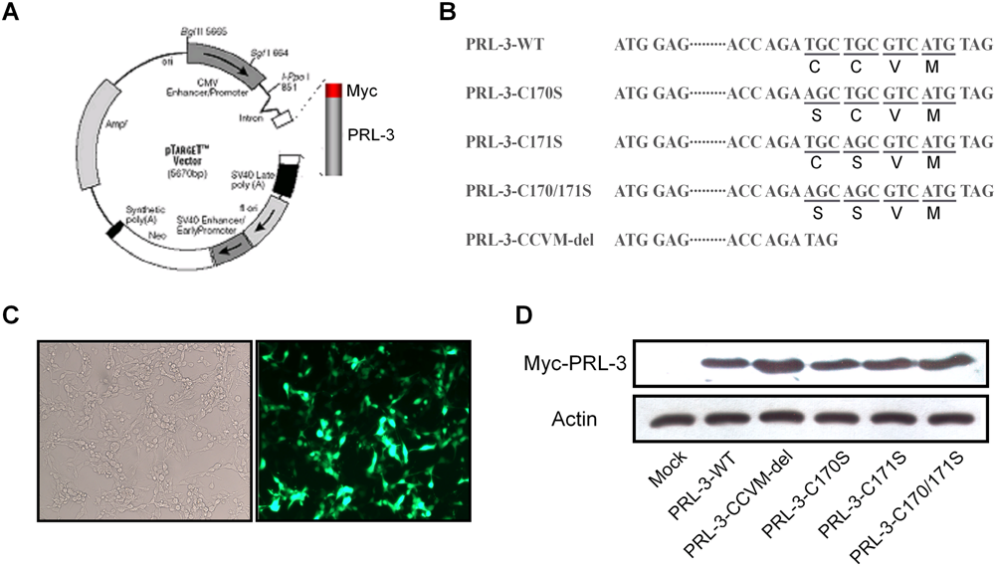
Construction of Myc-PRL-3 fusion expression vector and expression in B16F1 murine melanoma cells. (A) Schematic drawing of pTARGET-Myc-PRL-3 expression vector. (B) Schematic representation of Myc-PRL-3 mutations. Mutations were constructed from pTARGET-Myc-PRL-3-WT by PCR. (C) Photographs of transfection efficiency of B16F1 cells. B16F1 cells were transfected with pEGFP for 24 hours and then observed with fluorescent microscopy (right); same view as the right but with a light microscopy (left). (D) Level of Myc-PRL-3 was detected by Western blot. Results shown are representative of three independent trials.

To investigate the subcellular localization of PRL-3, we constructed wild-type (WT) and mutant Myc-PRL-3 fusion expression vectors. The mutant vectors consisted of mutations to the amino acids 170 to 173 of PRL-3 C-terminal CCVM motif, including Myc-PRL-3-C170S (C at 170 replaced with S), Myc-PRL-3-C171S (C at 171 replaced with S), Myc-PRL-3-C170/171S (both C at 170 and 171 replaced with S) and Myc-PRL-3-CCVM-del (without the CCVM structure in the C-terminal) (Fig. 1A and B). A high percentage of the B16F1 cells expressed GFP 24 hours after transient transfection with pEGFP-N1 indicating efficient transfection (Fig. 1C). Meanwhile, protein extracts of B16F1 cells transfected with PRL-3-WT or mutations were analyzed with c-Myc antibody by Western blot. As shown in Fig. 1D, Myc-PRL-3-WT and their mutations were efficiently expressed in B16F1 murine melanoma cells.

### Subcellular localization of PRL-3 with a critical site of 170 cysteine

**Figure 2:**
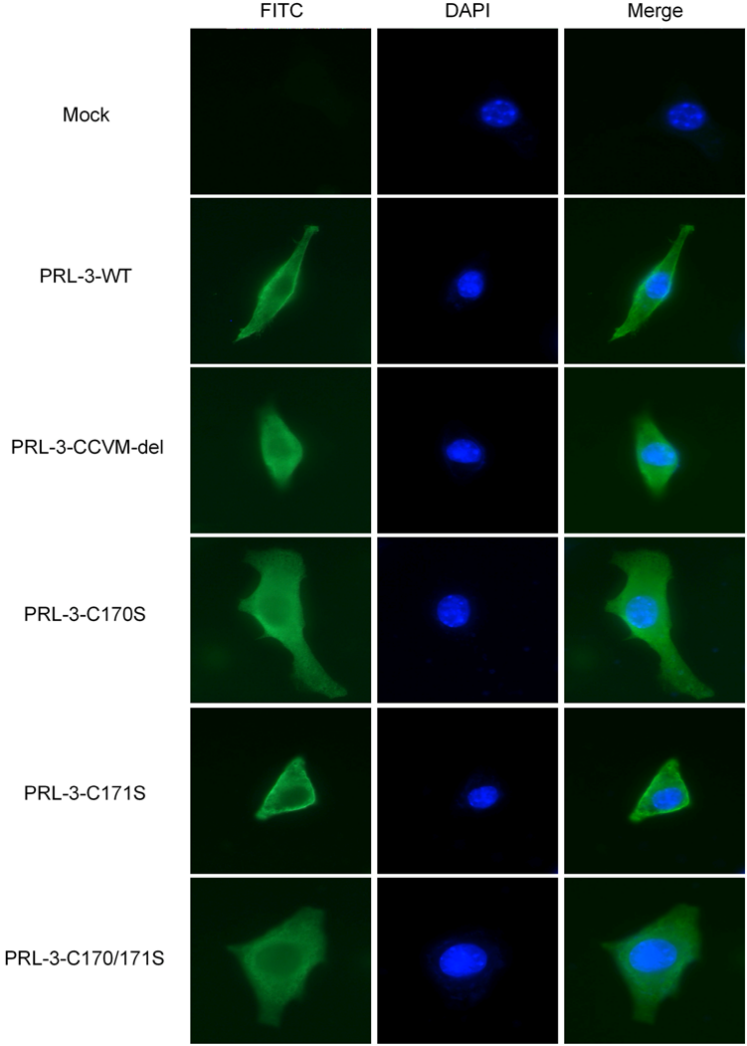
Intracellular localization of PRL-3. B16F1 melanoma cells were transiently transfected with wild type and mutated Myc-PRL-3 expression vectors. Twenty-four hours after transfection, cells were fixed with 4% paraformaldehyde and stained with anti-Myc primary antibody and FITC labeled secondary antibody. Myc-PRL-3 wild type and mutations were detected under fluorescence microscopy (400×). Nuclei were counterstained with 4, 6-diamidino-2-phemylindole (blue). Pictures shown are representative of three independent trials.

The subcellular localization of PRL-3 was observed by immunofluorescent staining with c-Myc antibody 24 hours following transient transfection of B16F1 cells with PRL-3-WT or mutation vectors. PRL-3-WT and PRL-3-C171S mutants were located on cytoplasmic membrane (Fig. 2). In contrast, PRL-3-CCVM-del was mostly found within the cytosol. Likewise, PRL-3-C170S and PRL-3-C170/171S were also localized to the cell cytosol.

### Increased adhesion and migration of B16F1 transfectant cells with relation to cysteine170 of PRL-3 and inhibition by FTI-277

**Figure 3:**
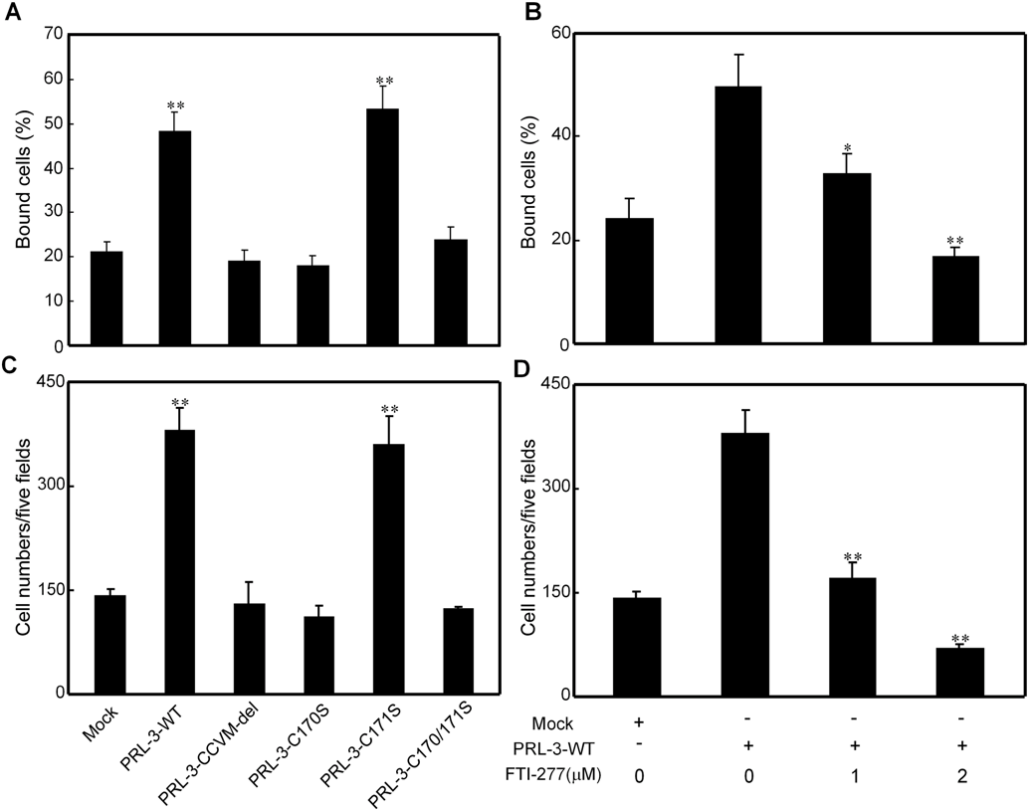
Effects of PRL-3-WT/mutations and prenylation inhibitor FTI-277 on B16F1 melanoma cells adhesion and migration ability. (A) B16F1 cells transfected with wild type or mutated Myc-PRL-3 expression vectors and (B) PRL-3-WT transfected cells pretreated with 1 µM or 2 µM FTI-277 for 18 hours were seeded into the wells of 96-well plates coated with fibronectin (10 µg/ml). After incubation for 30 minutes at 37°C, non-adherent cells were gently washed away. The amount of the adherent cells was determined at 592 nm after crystal violet staining. Data are mean±SEM of three independent experiments and each experiment includes triplicate sets. (C) A single-cell suspension (100 µl, 1×10^6^ cells/ml) of cells was plated into the upper wells of Transwell inserts containing 8 µm pore polycarbonate membranes pre-coated with fibronectin on the under surface. Cells were allowed to migrate for 24 hours at 37°C, and then fixed, stained with crystal violet and counted. Quantitative analysis of the number of the cells migrated to the lower side of the membrane is shown. (D) Treatment with FTI-277 inhibits the migration of B16F1 melanoma cells. B16F1 cells were transfected with PRL-3-WT and incubated with FTI-277 1 µM or 2 µM for 18 hours before plated into the upper wells of Transwell inserts, which still contain different concentrations of FTI-277. All Data are mean±SEM of three independent experiments. *, P<0.05; **, P<0.01, vs. mock vector for (A) and (C) and vs. PRL-3-WT expression vector for (B) and (D).

The adhesion capabilities of B16F1 cells were analyzed 24 hours following transfection with PRL-3-WT or the mutants. Thirty minutes after plating, the adhesion capability to fibronectin of B16F1 cells transfected with PRL-3-WT and PRL-3-C171S was significantly increased to 48% and 53%, respectively, compared with 21% adhesion of cells treated with mock vector (Fig. 3A). In contrast, transfection with PRL-3-CCVM-del, PRL-3-C170S and PRL-3-C170/171S had little effect on the cell adhesion to fibronectin (Fig. 3A). Similar to the results in adhesion assay, transfection of B16F1 cells with PRL-3-WT and PRL-3-C171S resulted in a 2.7- and 2.5-fold, respectively, enhanced migration to the under surface compared to cells transfected with mock vector 24 hours following plating (Fig. 3C). However, transfection with PRL-3-CCVM-del, PRL-3-C170S and PRL-3-C170/171S had no such effects on cellular migration (Fig. 3C).

The carboxy-terminal CCVM motif of PRL-3 belongs to a broad class of proteins containing a CAAX motif, which is often modified by post-prenylation. To determine the role of prenylation in PRL-3-induced cell adhesion and migration the farnesyltransferase inhibitor, FTI-277 was used. Pretreatment of B16F1 cells transfected with PRL-3-WT with FTI-277 (1 µM or 2 µM) for 18 hours of the 24 hour post transfection period resulted in a reduction of cell adhesion to 32% and 16% as compared with 50% of untreated PRL-3-WT transfected cells (Fig 3B). Likewise, the migratory capability of PRL-3-WT transfected B16F1 cells was decreased by 55% and 82% following FTI-277 (1 µM or 2 µM) treatment, respectively, as compared with untreated PRL-3-WT cells (Fig. 3D).

### Metastatic tumor formation of B16F1 cells transfected with PRL-3-WT or mutants

**Figure 4:**
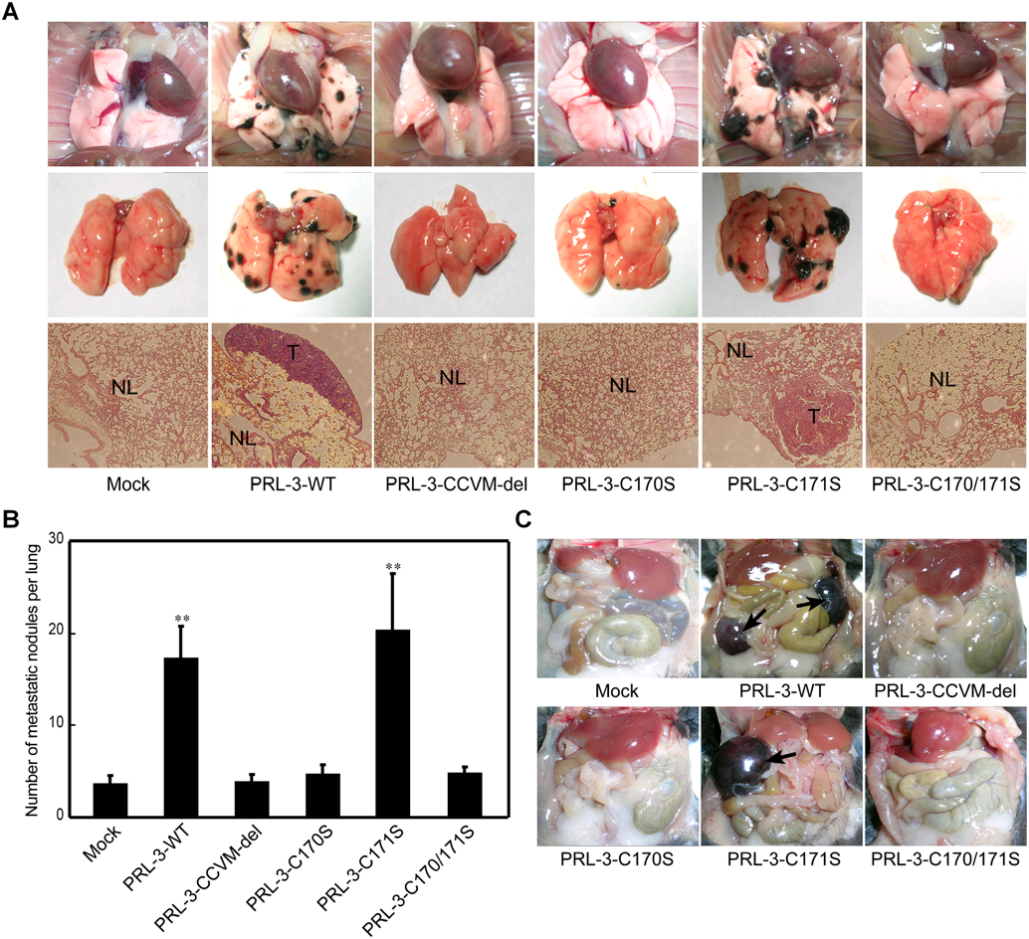
Effect of PRL-3-WT/mutations on B16F1 melanoma cells metastasis *in vivo*. B16F1 melanoma cells transfected with mock vector, wild type or mutated Myc-PRL-3 expression vectors were injected intravenously into C57BL/6J mice via tail vein. After 20 days, the mice were dissected and analyzed for metastasis. (A) Representative examples of lungs from the mice. The top and middle panels show the stereo micrographs of lungs. The lower panels show the histological photomicrographs of lung section stained with hemotoxylin and eosin (100×). NL, normal lung tissue; T, metastatic tumor lesions. (B) Quantitative evaluation of macroscopically detectable lung metastases. After fixation in Bouin's solution, the number of macroscopically visible metastases on the lung surface was quantified. (C) Representative example of mesentery lymph node metastatic site. Data are mean±SEM of six mice. **, P<0.01, vs. mock vector.

C57BL/6J mice were injected with B16F1 cells transfected with PRL-3-WT, mutants or mock vector (Fig. 4). Twenty days later, considerable pulmonary metastasis was found in mice injected with PRL-3-WT and PRL-3-C171S transfected cells, with average nodule number of 17 and 20, respectively (Fig. 4B), and severe tumor cell infiltration could be observed with histological examination (Fig. 4A). In contrast, only a slight pulmonary metastasis with an average nodule number of 4, 4, 5 and 5 was detected in mice injected with cells transfected with mock vector, PRL-3-CCVM-del, PRL-3-C170S or PRL-3-C170/171S, respectively (Fig. 4B). In these mice there was only alleviative tumor cell infiltration (Fig. 4A). In addition, two of six mice carrying PRL-3-WT transfected cells and three of six injected with PRL-3-C171S transfectants had metastatic lesions in both of the mesentery lymph nodes and the lung (Fig. 4C). No other metastatic sites were observed in liver, kidney or colon.

## Discussion

Our previous studies showed that the phosphatase activity of PRL-3 was involved in the promotion of tumor metastasis [3]. However, little is known about the role of PRL-3's cellular localization in this tumorigenic process. In order to explore the relationship between PRL-3's subcellular localization and its metastatic function, we utilized an over-expression system with Myc fusion protein. We amplified PRL-3 gene from mice skeleton muscle and constructed Myc-PRL-3 wild type and four mutant fusion expression vectors (Fig. 1A). The two cysteines of PRL-3 CCVM were mutated to serines (Fig. 1B) to specify the exact crucial site in tumor migration.

Proteins with CAAX motif are often farnesyled or geranylgeranyled, which adds a long hydrophobic fatty acid chain to the proteins and facilitates their plasma membrane localization [20]. Though various proteins of the CAAX family depend on such modifications for correct localization, they can be targeted to different subcellular sites [21], [22]. In our current study, with the effective transfection system shown in Fig. 1, PRL-3 wild type and the mutant vectors were introduced into B16F1 to determine the subcellular localization of PRL-3. As shown by immunofluorescent microscopy, wild type PRL-3 was localized to cell cyto-membrane, while the C170S mutation resulted in the diversion of the majority of the protein to the whole cytosol. However, such whole cytosol distribution did not happen in the cells transfected with PRL-3-C171S mutants. Although the specific subcellular localization of PRL-3 was not examined in this work, our current data suggested, at least that PRL-3 could locate on the plasma membrane of murine melanoma cells and further, Cys170 was the key site for its localization while Cys171 contributed little to this progress. These results may be inconsistent with some previous conclusions. For example, Fiordalisi et al. [18] transfected colon cancer cell line SW180 with GFP-PRL-3 fusion protein and found PRL-3 located on the endoplasts and recticulum membrane while its SAXX mutants were observed in the nucleus. Zeng et al. [11] reported that over-expression of GFP-PRL-3 fusion protein in CHO cells resulted in protein localization to certain regions of plasmic membrane such as membrane ruffles, protrusions and some vacuolar-like membrane extensions. These differences could be explained by cell type or fusion protein system specificity mediating localization due to differences in cell signaling and protein interaction. In previous studies [11], [18], the 22 kDa PRL-3 was fused to GFP of 27 kDa [23], facilitating localization tracing, but possibly inducing side effects on the PRL-3's localization in normal status. Since GFP was bigger than PRL-3 in molecular size, it might produce steric hindrance for correct localization of PRL-3. The PRL-3's localization observed in our study could be supported from the reports of Bardelli et al. [2] in which over expression of HA-PRL-3 in colon cancer cells CRC resulted in cell plasmic membrane localization of PRL-3. It still needs to be determined whether PRL-3 would localize to other membrane structures within the cell.

We then determined the role of PRL-3 prenylation on the cancer cell adhesion and migration ability *in vitro*. Compared to the control group transfected with mock vector, PRL-3 transfectants with PRL-3-WT and PRL-3-C171S had an elevated ability of adhesion and migration to strata of fibronectin (Fig. 3A and C). In contrast the other two mutants, PRL-3-C170S and PRL-3-C170/171S, had no effect on the rate of adhesion or migration. We thus hypothesize that PRL-3 localizing to cell cyto-membrane is indispensable for its function in cancer metastasis via participation in the signal transduction pathway on the inner side of the membrane. In fact, by using a yeast two-hybrid system to identify integrin α1 on cell membrane as a PRL-3-interacting protein, Peng et al. [24] demonstrated that PRL-3 could down-regulate the tyrosine-phosphorylation level of integrin β1, hence activating the MAPK pathway. Furthermore, we found that C170S mutants showed a strongly reduced ability of cell adhesion and migration, and treatment with the prenylation transferase inhibitor FTI-277 also decreased the ability of PRL-3-WT transfected B16F1 cells (Fig. 3B and D). This result supported a model of Cys170 prenylation in normal PRL-3 function. At the same time, we found that when treated with high concentration (2 µM) of FTI-277, PRL-3-WT transfected B16F1 cells showed even lower migration ability compared with mock vector, suggesting that FTI-277 might inhibit some other molecules' function. In fact, the most proteins of the CAAX family are also oncogenes, such as Ras and Rho super-family [14], [25]. For this reason, investigations into the mechanisms of farnesylation and the drug-screening for prenylation transferase inhibitors are becoming a new hotspot of pharmacology. A number of such inhibitors are already in phase III clinical trials as potential new generation of agents for anticancer treatment with their low toxicity and high efficiency [26]. Our current study offers PRL-3 as a new target for this kind of medicine treatment.

Since tumor metastasis is a highly complex, multi-step progress, the *in vivo* behavior of wild type PRL-3 and its mutation transfectants' were further examined. In an experimental passive tumor metastasis model, we found that few nodules were formed in the lung of mice injected by mock vector transfectants. Such pulmonary metastasis was apparently increased in those seeded by PRL-3-WT and PRL-3-C171S mutants. In contrast, mice with the other mutant-transfected B16F1 showed low tumorigenic behavior similar to the control group (Fig. 4A and B). In addition, apparent mesentery lymph node metastatic lesions were also observed in mice injected with PRL-3-WT and PRL-3-C171S mutants (Fig. 4C), but not those with mock vector, PRL-3-CCVM-del or the other two mutants. These results further support our hypothesis that PRL-3's localization to plasma membrane directed by Cys170 prenylation is crucial for its function in promoting tumor metastasis.

In summary, our current study demonstrated the critical role of CCVM motif for PRL-3 intracellular localization and promotion of tumor metastasis, of which Cys170 was the key amino acid for prenylation. The localization of PRL-3 to cell cyto-membrane was highly correlated with its function in the progress of metastasis, shedding some light for further investigation on its signal transduction pathway. Prenylation transferase inhibitors, which showed an effective inhibition on cancer cell migration, also suggested a promising future for the selection of such drugs as anti-cancer agents.

## Materials and Methods

### Cell culture

Lowly metastatic cell line B16F1 cells were maintained in DMEM (Dulbecco's modified Eagle's medium, Life Technologies Inc., Grand Island, NY) supplemented with 10% FBS (fetal bovine serum, Life Technologies Inc.), 100 U/ml penicillin, and 100 µg/ml streptomycin, and incubated at 37°C in a humidified atmosphere containing 5% CO_2_ in the air.

### Animals

C57BL/6J mice (6 to 8 weeks old) were obtained from the Shanghai Laboratory Animal Center (Shanghai, China). Throughout the experiments, mice were maintained with free access to pellet food and water in plastic cages at 21±2°C and kept on a 12-hour light-dark cycle. Animal welfare and experimental procedures were performed strictly in accordance with the “Principles of laboratory animal care” (NIH publication No. 86-23, revised 1985) and the related ethical regulations of China. All efforts were made to minimize the animals' suffering and to reduce animal consumption.

### Construction of vectors and transient transfection in cells

Myc-PRL-3-WT was generated by RT-PCR with the following primers: sense, 5′-GCGGATCCACCATGGAGCAGAAGCTGATCTCCGAGGAGGACCTCGCCCGCATGAACCGGCCTGCGCCTG-3′ and antisense, 5′-CTGGTACCCTACATGACGCAGCATCTGGTC-3′ from muscle total RNA of C57BL/6J mouse. And mutants were constructed using different antisense primers: Myc-PRL-3-C170S: 5′-CTGGTACCCTACATGACGCAGCTTCTGGTCTTGTGCGTGTG-3′; Myc-PRL-3-C171S: 5′-CTGGTACCCTACATGACGCTGCATCTGGTCTTGTGCGTGTG-3′; Myc-PRL-3-C170/171S: 5′-CTGGTACCCTACATGACGCTGCTTCTGGTCTTGTGCGTGTG-3′; Myc-PRL-3-CCVM-del: 5′-CTGGTACCCTATCTGGTCTTGTGCGTGTG-3′. Then, the PCR fragments were cloned into BamH I/Kpn I sites of pTARGET (Promega).

Transient transfections were performed to transfer vectors to B16F1 murine cells. Briefly, 4 µl lipofectamine 2000 (Life Technologies Inc., Grand Island, NY) and 2 µg plasmids were diluted in 50 µl MEM (Life Technologies Inc., Grand Island, NY) and mixed, then incubated at room temperature for 20 minutes before addition of 900 µl MEM. The mixture was spread onto cells. Cells were switched to 10% FBS DMEM after 6 hours and incubated at 37°C for another 18 hours, then collected and prepared for the following experiments.

### Western blot

Western blot assay was performed as described [27]. The B16F1 cells transfected with mock vector, Myc-PRL-3-WT, Myc-PRL-3-CCVM-del, Myc-PRL-3-C170S, Myc-PRL-3-C171S and Myc-PRL-3-C170/171S were collected and lysed (50 mM Tris pH 8.0, 150 mM NaCl, 1% NP-40, 0.1% SDS, 5 mM EDTA, 0.1 mM PMSF (phenylmethylsulfonylfluoride), 0.15 U/ml aprotinin, 1 µg/ml pepstatin and 10% glycerol). Anti-Myc (clone 9E10) and anti-actin (Santa Cruz Biotechnology) antibodies were used for the assay.

### Immunofluorescence microscopy

B16F1 cells transfected with PRL-3-WT or mutants for 24 hours on glass cover slips were washed with PBS (phosphate-buffered saline) and fixed with 4% paraformaldehyde on ice for 30 minutes, followed by permeabilization with 0.1% Triton X-100 for 30 minutes. Cells were blocked with PBS containing 10% serum for 1 hour at room temperature and then incubated with c-Myc antibody overnight at 4°C. After washed three times with PBS, cells were incubated with anti-mouse IgG conjugated with FITC (Sigma) for 1 hour at room temperature. The expression of fusion proteins was detected by fluorescence microscope. Nuclei were counterstained with 2 g/ml 4,6-diamidino-2-phenylindole (Sigma).

### Cell adhesion assay

Cell adhesion assay was performed essentially as described [28] with some modifications. In brief, 96-well flat-bottom plates were coated with 50 µl fibronectin (10 µg/ml; Sigma) in PBS overnight at 4°C and then blocked with 0.2% BSA for 2 hours at room temperature followed by three times wash. Next, B16F1 cells, transiently transfected with PRL-3-WT and mutants for 24 hours, were added into each well in triplicate and incubated for 30 minutes at 37°C. For FTI-277 (Calbiochem) treatment group, cells were pre-incubated with FTI-277 for 18 hours. Plates were then washed three times with PBS to remove unbound cells. Cells remained attaching to the plates were fixed and stained with a solution containing 0.5% crystal violet and 2% ethanol in 100 mM borate buffer (pH 9.0). After washing, 100 µl SDS (1% w/v) was added and the absorbance of the color substrate was measured with an ELISA reader (TECAN, Austria) at 592 nm. After subtraction of the background cell binding to BSA-coated wells, the percentage of adherent cells was calculated by dividing the optical density of the adherent cells by that of the initial input cells.

### Cell migration assay

Cell migration assay was performed using 8.0-µm pore size Transwell inserts (Costar Corp., Cambridge, MA) as described previously [3] with some modifications. In brief, the under surface of the membrane was coated with fibronectin (10 µg/ml) in PBS at 37°C for 2 hours. The membrane was washed in PBS to remove excess ligand, and the lower chamber was filled with 0.6 ml DMEM with 10% FBS. Cells were serum-starved overnight (0.5% FBS), harvested with trypsin/EDTA, and washed twice with serum-free DMEM. Then cells were re-suspended in migration medium (DMEM with 0.5% FBS), and 1×10^5^ cells in 0.1 ml were added to the upper chamber. For FTI-277 (Calbiochem) treatment group, cells were pre-incubated with FTI-277 for 18 hours and then added to the upper wells of Transwell inserts, which still contain FTI-277. After 24 hours at 37°C, the cells on the upper surface of the membrane were removed by cotton tips. The migrant cells attached to the lower surface were fixed in methanol at room temperature for 30 minutes, and stained for 20 minutes with a solution containing 0.5% crystal violet and 2% ethanol in 100 mM borate buffer (pH 9.0). The number of migrated cells on the lower surface of the membrane was counted under a microscope in five fields (100×).

### Tail Vein Metastasis Assay

After B16F1 cells were transiently transfected with PRL-3-WT and mutants, C57BL/6J mice were injected intravenously with 2×10^5^ cells in 0.1 ml PBS via tail veins. After 20 days, the mice were euthanized, and their lungs were resected and photographs were taken (Nikon Coolpix 4500) before fixation in Bouin's solution for further analysis. The numbers of metastatic nodules on the surface of the organs were counted macroscopically.

### Histology

Tissues were fixed in Bouin's solution for 24–48 hours. After washing with fresh PBS, fixed tissues were dehydrated, cleared, and embedded in paraffin (Paraplast regular, Sigma). Sections (5 µm) were collected on microscope slides, deparaffinized, and stained with H & E as routine procedures.

### Statistical analysis

Data were expressed as mean±SEM. Student's *t*-test was used to evaluate the difference between two groups. P<0.05 was considered to be significant.
